# Successes and Challenges in Clinical Trial Recruitment: The Experience of a New Study Team

**DOI:** 10.3390/medsci12030039

**Published:** 2024-08-14

**Authors:** Man Hung, Amir Mohajeri, Konstantinia Almpani, Gabriel Carberry, John F. Wisniewski, Kade Janes, Brooklyn Janes, Chase Hardy, Golnoush Zakeri, Ben Raymond, Heather Trinh, Jordan Bretner, Val J. Cheever, Rafael Garibyan, Perry Bachstein, Frank W. Licari

**Affiliations:** 1 College of Dental Medicine, Roseman University of Health Sciences, South Jordan, UT 84095, USA; 2 Division of Public Health, University of Utah, Salt Lake City, UT 84108, USA; 3 College of Engineering, University of Utah, Salt Lake City, UT 84108, USA; 4 Department of Educational Psychology, University of Utah, Salt Lake City, UT 84108, USA; 5 Huntsman Cancer Institute, Salt Lake City, UT 84112, USA; 6 Department of Veterans Affairs Medical Center, Salt Lake City, UT 84148, USA

**Keywords:** recruitment strategy, clinical trial, experiential learning, research education

## Abstract

Participant recruitment is one of the most challenging aspects of a clinical trial, directly impacting both the study’s duration and the quality of its results. Therefore, reporting successful recruitment strategies is crucial. This study aimed to document the recruitment tactics and experiences of a research team during a university-based randomized clinical trial, conducted as part of a clinical research immersion program. Recruitment took place from October 2021 to October 2022. Before the study commenced, study team members received formal training in clinical trial participant recruitment from the Principal Investigator. The recruitment strategies were integrated into initial study design, which was approved by the Institutional Review Board. A multimodal approach was employed, incorporating both direct and indirect recruitment methods. These strategies successfully met the enrollment target within the twelve-month period. Throughout the process, team members acquired valuable knowledge in recruitment design and implementation, along with transferable interpersonal and networking skills. In-person recruitment was the most efficient and cost-effective strategy, followed by personal referrals. The primary challenge was accommodating participants’ availability. Other study teams should consider these recruitment strategies during their study designs. Additionally, the knowledge and skills gained by this study team underscore the value of experiential learning in research education.

## 1. Introduction

One of the most challenging aspects of clinical trials is the successful recruitment and retention of participants based on the study’s target enrollment within the defined time frame. Failure to recruit the required number of eligible participants willing to commit to a clinical study can result in significant delays, financial losses, or reduced validity of the study results [1]. Furthermore, a low accrual rate is a leading cause of the termination of clinical trials [2,3,4]. Therefore, designing an effective recruitment strategy is vital for the successful conduct of any clinical trial.

Many methods for recruitment have been previously tested and reported in the literature, particularly for randomized clinical trials (RCTs) [5,6]. Strategies include increasing potential participants’ awareness of the health problem being studied [7,8], using advertising and social media to reach potential participants [9,10,11,12], in-person recruitment [13], offering incentives for participation [14,15], simplifying the enrollment process, providing clear and thorough information about the trial [6,16,17,18], and providing advance notification for study visits [15,19]. These methods have shown varying degrees of success.

However, the effectiveness of many of these strategies remains unclear [6], and there is no consensus on a specific plan that guarantees successful recruitment [20,21]. This variability is likely due to differences in the participant population [22], the nature of the study intervention and relevant risk, and the organization of the research study teams. Moreover, despite the importance of this issue, there is often a lack of reporting on the effective recruitment strategies in the publications associated with RCTs [4]. Sharing this information can improve the efficiency of future RCTs and other clinical trials, which provide the most reliable evidence for evaluating the effects of healthcare interventions.

This study aimed to report the experience of a research study team with recruitment strategies implemented for conducting a double-blind, randomized clinical trial on the effects of an oral probiotic intervention in oral health in a university dental college setting. Additionally, the value of this experience was evaluated as part of a clinical research immersion program integrated into this study.

## 2. Methods

### 2.1. Study Design

The present study described the training activities, evaluation of knowledge acquisition, recruitment efforts and outcomes of a new study team in conducting a double-blind randomized prospective clinical trial to investigate the effects of an oral probiotic intervention on oral health. Participants attended a university-based dental clinic every two weeks for five consecutive appointments for data collection and evaluation of oral healthcare product usage. Recruitment took place between 26 October 2021 and 25 October 2022. The clinical trial was approved by the Institutional Review Board of the university and registered on https://clinicaltrials.gov.

### 2.2. Training Activities

To develop training activities for teaching the new study team of faculty, staff, and students without clinical trial experience, the Principal Investigator employed a structured methodology that included both theoretical and practical components. The training program consisted of didactic lectures covering fundamental topics such as clinical trial design, ethical considerations, regulatory requirements, data management, good clinical practice, participant recruitment strategies, persuasive speaking, negotiation, participant consent, information technology, graphic design, marketing, and effective communication skills. Simultaneously, interactive components provided hands-on experience through mock trials and role-playing scenarios to simulate real-world challenges. This simultaneous approach ensured that didactic lectures were supplemented by interactive components to enhance understanding.

Mentorship and supervision were integral to the program, with the Principal Investigator providing ongoing guidance and support throughout the clinical trial. Regular weekly check-ins with faculty, staff, and students ensured that they could address questions and overcome difficulties. Feedback sessions conducted at least twice per week ensured everyone was aligned in their understanding of how to conduct the clinical trial and how to solve unexpected problems.

### 2.3. Evaluation of Knowledge Acquisition

To evaluate the acquisition of knowledge, the Principal Investigator implemented practical assessments focusing on real-world applications. Initially, each team member participated in mock trials and role-playing scenarios. The Principal Investigator observed these sessions, providing immediate feedback and guidance. Team members repeated these exercises until they demonstrated proficiency in necessary skills. During the actual clinical trial, the Principal Investigator used a checklist to monitor each member’s performance in their assigned roles. This continuous, weekly evaluation ensured that all team members consistently met the required standards and effectively contributed to the trial.

### 2.4. Study Team

The study team consisted of forty-four members, including seven faculty, six staff, and thirty-one students. Most team members had minimal or no prior experience in clinical research, but all received human subject research certification and good clinical practice certification before the study began.

### 2.5. Recruitment Strategy

A thorough literature search was conducted to identify effective clinical trial recruitment methods. Based on the information acquired, a recruitment strategy was strategically designed before the beginning of the trial. All study team members participated in the recruitment strategy design process. A multimodality approach was developed, with the implementation of a variety of recruitment methods that were used concurrently. These methods include in-person recruitment, advertising in the form of fliers, university-wide emails, and social media postings, direct referrals from study participants, and participation in dental college community service events.

### 2.6. In-Person Recruitment

Based on the results of previous studies, recruiting participants in person has been proven successful/efficient [23,24,25,26] and cost-effective [27,28,29]. The study’s Principal Investigator conducted extensive formal training for all research team members before the beginning of the trial on recruiting techniques/approaches, emphasizing the importance of respecting potential participants’ privacy without undue pressure while providing an accurate and unbiased description of the study. In-person recruitment mainly occurred within the university, among peers, faculty members, staff, and clinic patients. Through clear communication, research study team members were able to approach potential participants and provide adequate information about the study’s objective and the participants’ requirements, while also taking the opportunity to answer questions. In addition, the positive benefits of enrolling in the study were discussed. These aspects included the minimal risk of the intervention, monetary compensation, and a complementary dental cleaning at the dental college clinic.

### 2.7. Fliers

A flier was created to include essential information about the study and contact details for those interested in participating. Text, photos, and graphics were combined to make it more attractive and effective. The flier focused on providing information about the study, including eligibility, purpose, scheduling, and duration. Furthermore, it provided contact information such as an email address and phone number specifically created for the research.

The flier was utilized in both digital and printed format. The digital version was posted on social media accounts and online advertising platforms. Study team members distributed printed fliers to local businesses, religious organizations, and neighboring communities after acquiring permission. Printed fliers were also continuously placed at the patient check-in locations and waiting rooms to inform patients and their companions about the study. An enlarged version of the flier was posted on the main dental college announcement boards for added visibility.

### 2.8. Referrals

Direct referrals from study team members were beneficial due to the large number of students involved in the study. These student team members continuously promoted the study and directly invited their colleagues, family, and friends to participate. Referrals from study participants were systematically requested as part of the study visit procedures. Team members were trained with specific vocabulary and techniques to appropriately solicit participants’ feedback during the final visit. Based on their responses, whenever there was positive feedback, study team members would take the opportunity to encourage the participants to invite friends, family members, and co-workers to join the study. Participants were motivated to share their positive experience related to the study and were also provided with printed fliers that they could pass on to those who would be interested in more details and contact information.

### 2.9. Community Service Event

During a scheduled university community service event where dental students provided free dental care to children in need, the study team partnered with the event’s organizing committee and set up a booth for participant recruitment. Team members used this opportunity to further advertise the study and engage with the local community. Study team members were strategically stationed to ensure that the incoming visitors could easily view the booth. A large banner and a color wheel with small prizes were placed next to the table to attract additional attention. Team members actively engaged visitors by providing information about the study, answering questions, and emphasizing the low risk of the intervention, the significant monetary incentive, and the complimentary dental cleaning appointment included in the study.

### 2.10. Social Media

Information about the clinical trial was regularly posted by study team members and displayed on various social media platforms, including the official social media pages of the university and dental college, dental professional group pages, and in university and dental college email newsletters. Additionally, an advertisement for the study was placed on a popular classified city-wide advertising platform, which required a monthly subscription.

### 2.11. Procedure

Participants interested in the study or who had additional questions contacted the team via phone or email using the information provided in the study flier. A study team member responded promptly and scheduled screening appointments for interested individuals. Team members regularly checked messages and voicemails to ensure timely responses. All communications with participants throughout the study were recorded.

## 3. Results

As a result of implementing the current recruitment strategies, the study successfully achieved its enrollment target within the initially estimated timeframe of twelve months. The study team members acquired valuable knowledge and skills during the recruitment process. In-person recruitment was the most successful recruitment method, with 81 subjects prescreened and all 46 who were screened completing the study, yielding a 100% completion rate (Table 1). Through the in-person recruitment process, team members have gained valuable insights into research subject recruitment and enhanced their interpersonal and networking skills.

Additional networking training opportunities were created for the study team members through their interactions with the participants. As a result, most participants in the study had a positive experience. Following this observation, research team members began using participant referral requests to recruit new participants. This method was implemented during the participants’ final study visit. Ensuring that participants had a positive overall experience and directly involving them in recruitment efforts has been a critical element for successful recruitment in this study. The referral strategy was highly effective, with 37 individuals prescreened, 19 screened, and all 19 completing the study, resulting in a 100% completion rate (Table 1). This observation highlights that participant satisfaction is crucial for their retention in the study, the overall quality of the final results, and perpetuating a successful recruitment process.

Using fliers as part of the recruitment strategy was shown to be effective as well. Both printed and digital versions were widely utilized and demonstrated significant usefulness. These fliers reached a broader audience beyond the social networks of study team members and participants. As a learning experience, team members gained valuable insight into research marketing strategies and advertising tactics. During the prospective patient prescreening, many potential participants were drawn to learn more about the study due to the information provided in the flier and the monetary compensation for participation. Optimizing the flier design based on the participant feedback was also a valuable experience for the study team members, highlighting the necessity for ongoing assessment and dynamic adaptation of recruitment methods. The updated version of the flier was more effective in capturing the attention of new participants, and was utilized until the completion of the trial. The flier strategy resulted in 63 individuals prescreened, 23 screened, and 22 completing the study, yielding a completion rate of 95.7% (Table 1).

Subject recruitment through social media was also very effective. University-wide emails and social media platforms allowed information about the study to reach the entire university community. All study team members collaborated to ensure constant study coverage on social media. Additionally, advertising the study on a public website informed potential participants with no previous connection to the university or dental college about the study. As a result, many individuals contacted the study team, expressing interest in participating. The social media strategy prescreened 102 individuals, with 13 screened and 12 completing the study, resulting in a completion rate of 92.3% (Table 1).

The university community service event also demonstrated to be a successful recruitment method. However, due to the ethnic consistency of the population attending the event, language barriers between study members and potential participants were a significant drawback on many occasions. Nevertheless, it provided an additional opportunity for study team members to practice their interpersonal communication skills and learn how to adapt their language to describe the study. Utilizing layperson’s terms to ensure effective communication was vital in engaging with potential participants. The community service event strategy prescreened 34 individuals, with eight screened and six completing the study, resulting in a completion rate of 75% (Table 1).

### Challenges

Many challenges were encountered during the recruitment process. Initially, issues related to the organization of the study team and common participant-related problems arose. Specifically, there was a limited number of team members, and few felt confident in their ability to contribute to recruitment efforts. However, these issues were promptly resolved by adding more members to the team and providing additional training on subject recruitment. The study’s Principal Investigator also offered encouragement, which helped boost team members’ confidence and effectiveness in recruitment.

Challenges related to the participants included scheduling conflicts that prevented them from attending study visits on the specific days and times the study clinic was operating, as well as failures to attend screening appointments. To overcome these challenges, efforts were made to accommodate the schedules of eligible subjects to ensure their participation in the study. Additionally, reminders were sent more consistently to new participants to confirm their attendance for the screening process.

Finally, partnering with local organizations to distribute study fliers was challenging at times. While many local businesses were willing to participate, some facilities cited liability issues as a reason for not participating in our recruitment efforts. However, this problem did not significantly hinder the research team’s recruitment efforts. Team members adapted by implementing additional previously mentioned strategies to compensate for this obstacle.

## 4. Discussion

The recruitment experience gained by the research study team in this clinical research immersion program was substantial, and positively impacted each team member’s academic and career prospects. Additionally, the research team successfully recruited the targeted number of participants within the twelve-month timeframe, significantly contributing to the clinical trial’s success. The experiences reported here highlight effective recruitment strategies and the challenges encountered during the process.

An important facilitator of recruitment success was the development of a multimodal recruitment approach, strategically designed before the trial began. This approach was based on evidence-based methods previously reported in the literature [4,10,27]. Factors considered in selecting and utilizing these techniques included the characteristics of the study population and the working dynamics of the research team. Combining various recruitment strategies increased the likelihood of a steady inflow of new patients, as some methods could be more effective than others.

In addition, sharing recruitment responsibilities among all study team members enhanced the recruitment efforts. Assigning these responsibilities to specific members would not have been as productive. Moreover, receiving feedback from study members on the effectiveness of particular strategies was very useful. This feedback was part of the Six Sigma continuous improvement process and qualitative evaluation of the implemented strategies. When necessary, adapting based on this feedback led to optimizing the recruitment process.

The active involvement of team members in all aspects of the recruitment phase served as a valuable learning experience for each individual. As a result, members gained new knowledge in clinical research recruitment strategy design, implementation, and communication with research subjects. Additionally, they further developed their interpersonal communication and marketing skills. The knowledge and skills acquired are transferable to other aspects of their careers, and can be effectively gained through experiential learning, such as active participation in this study.

In-person recruitment was the most successful strategy, both in terms of recruitment numbers and cost-effectiveness. The effectiveness of this technique is consistent with other studies that have previously reported similar results regarding in-person recruitment [23,24,25,26,27,28,29]. This strategy was particularly successful in this study because the clinical trial was university-based, with abundance of dental students and students from three other healthcare-related academic programs nearby. An additional advantage was that a large portion of the subjects were dental students. This group of individuals was more compliant in attending their clinical trial visits since they were already at the location and had a heightened interest in oral hygiene, making them more likely to adhere to the trial rules.

Despite the increasing use of social media for recruitment purposes in medical research, prior studies comparing its effectiveness and efficiency with more traditional recruitment methods are limited [30]. Based on our clinical trial experience, advertising through social media was very effective for recruitment and significantly helped in reaching a broader segment of the population. Although dental students showed increased interest in participating, the study was open to the public, facilitating the swift enrollment of participants.

Referrals from study team members, who asked participants to invite friends, family members, and co-workers during the participants’ last appointment in the research clinic, were another successful strategy. The importance of this technique has been reported in previous studies, and was successfully implemented in this study [31]. In this case, a critical factor is ensuring that the participants have a positive experience as trial subjects. That includes effective communication with the study team members, good scheduling and punctuality of the appointment times, and professionalism of the study team in all aspects of the study. These elements will build a trusting relationship between the participants and the study team and motivate them to assist with their personal referrals. To provide context for our recruitment efforts, we compared our findings to other randomized clinical trial studies. We were able to successfully reach our recruitment targets without requiring extensions or additional funding. A systematic review found that 76% randomized clinical trials were discontinued due to poor recruitment [4]. Another systematic review found that only 55% of trials met their originally specified recruitment targets, reflecting considerable challenges in participant retention and completion [32]. Our successful completion of the randomized clinical trial indicates that our recruitment strategies and our Principal Investigator’s guidance were highly effective and could serve as a model for similar studies.

Finally, the significant financial compensation offered upon completion of the study was a noteworthy incentive for the recruitment of new participants and the retention of the enrolled participants. Although this is not information that the participants have explicitly reported, this is what the experience of the study team members indicated based on patients’ responses. In addition, reports on recruitment from prior studies [33,34] also emphasize the importance of compensation as a significant incentive.

Common problems and challenges to recruitment were reported, with the most prevalent problem being related to the clinic where participants were being visited. Although the time allotted to recruiting did not pose an issue due to the large, well-calibrated team, finding participants who were available during clinic hours did pose a challenge. The clinic used to conduct the study was only made available one morning and one afternoon a week. Thus, a total of eight hours a week of clinic time was allotted by the university. This schedule could not be easily altered once the study time slots were assigned. The limited clinic availability posed a complex problem for various subjects, such as employment or academic obligations conflicting with the clinic appointment times. To overcome this challenge, additional emphasis was given to effectively communicating with the participants about the importance of attending the study appointments regularly and confirming them in advance. A reminder system was implemented, with email and text reminders that were sent out to all participants one day before their scheduled appointments. Lastly, a review of previous interventional trials has shown that the mass mailing of brochures, flyers, and letters was effective in recruiting participants into these trials [35].

The lack of prior experience in conducting clinical trials at the institution also posed a barrier to recruitment. When staff do not view clinical trials as essential, it can create a detached research culture and adversely impact recruitment outcomes [36,37,38]. Moreover, the limited availability of team members for participant recruitment at the beginning of the study negatively impacted the initial enrollment rates. Nevertheless, this issue was quickly resolved with the inclusion of more study team members. In addition, students who were enrolled in a dental college became research team members, and were of great assistance throughout the recruitment phase.

The trend of new healthcare professional schools opening in the United States is increasing. Faculty and students in these new schools may have minimal or no research experience in conducting patient recruitment for clinical trials. At the startup stage, these schools typically prioritize establishing basic science and pre-clinical curricula, transitioning to clinical courses, and providing patient care. Patients will primarily be recruited for receiving treatment from students rather than for participating in clinical research. Once these newer schools become more established, incorporating a research component may become the administration’s next focus. Older schools with ongoing research have undergone this process, recognizing that it takes time to implement clinical trials and establish a successful research program. The experiences in patient recruitment for this clinical trial, which led to a successful outcome, have been well-documented by our research team. These recruitment strategies and techniques can serve as a “recipe for success” for new healthcare professional schools as they venture into the research arena.

The findings of this study on recruitment methods, while derived from a specific university context, have broader implications for diverse settings. The effectiveness of in-person recruitment, fliers, referrals, community service events, and social media can be adapted to various environments such as hospitals, community clinics, and other academic institutions. For instance, the high completion rates observed in in-person and referral strategies suggest that personalized approaches and leveraging existing networks can be universally effective. Similarly, social media’s broad reach highlights its potential in engaging a larger, more diverse population. By tailoring these strategies to the unique demographics and resources of different settings, other institutions can enhance their recruitment efforts.

### Strengths and Limitations

This study benefited from being conducted within a university dental college, where 400 dental students, along with faculty and staff, had easy access to the clinic. Word of mouth was an effective and straightforward technique for recruiting patients for this clinical trial. Additionally, having a large number of calibrated team members was vital for the efficiency and scheduling availability of the research clinic. Providing maximal flexibility in scheduling time slots for patient participation was also crucial during the recruitment process.

The major limitation of the results is that the reported strategies are based on the perceptions of the study members which, however, matched the objective findings seen in Table 1. Future studies could more systematically evaluate recruitment strategies by incorporating a robust experimental design that includes randomization and control groups. By assigning different recruitment methods to distinct participant groups and comparing the outcomes, researchers can better isolate the effects of each strategy. Additionally, employing mixed-methods approaches that combine quantitative data, such as the number of participants prescreened, screened, and completed, with qualitative feedback from participants can provide deeper insights into the reasons behind the effectiveness of each method. Utilizing advanced analytics, such as predictive modeling and machine learning, can also help identify patterns and optimize recruitment strategies. Regularly updating and integrating electronic medical records and scheduling software can streamline the identification and tracking of eligible participants. By employing these systematic approaches, future studies can more accurately assess and refine recruitment strategies to enhance participation rates and study outcomes.

## 5. Conclusions

This study highlights successful recruitment strategies and challenges encountered during participant recruitment. The findings indicate that successful recruitment in clinical trials requires a carefully designed, multimodal strategy tailored to the target population and the specific needs of the study. Reviewing these recruitment challenges provides researchers with opportunities to make adjustments and develop effective plans for recruiting similar participant samples. Future clinical researchers need to understand why recruitment challenges persist and apply these lessons to improve participant recruitment in future studies. We hope that health professional schools such as medical and dental schools, especially new ones, can implement similar strategies used in this study to advance their research efforts.

## Figures and Tables

**Table 1:** Number of participants recruited using different strategies.

Strategies	Number Prescreened	Number Screened	Number Completed the Study
In-person	81	46	46
Fliers	63	23	22
Referrals	37	19	19
Community Service Event	34	8	6
Social Media	102	13	12
Total Participants	317	109	105

## Data Availability

The original contributions presented in the study are included in the article. Further inquiries can be directed to the corresponding author.
